# Dynamic creation of a topologically-ordered Hamiltonian using spin-pulse control in the Heisenberg model

**DOI:** 10.1038/srep10076

**Published:** 2015-06-17

**Authors:** Tetsufumi Tanamoto, Keiji Ono, Yu-xi Liu, Franco Nori

**Affiliations:** 1 Corporate R & D center, Toshiba Corporation, Saiwai-ku, Kawasaki 212-8582, Japan; 2 Low temperature physics laboratory, RIKEN, Wako-shi, Saitama 351-0198, China; 3 Institute of Microelectronics, Tsinghua University, Beijing 100084, China; 4 CEMS, RIKEN, Wako-shi, Saitama, 351-0198, Japan; 5 Tsinghua National Laboratory for Information Science and Technology (TNList), Beijing 100084, China; 6 Physics Department, The University of Michigan, Ann Arbor, Michigan 48109-1040, USA

## Abstract

Hamiltonian engineering is an important approach for quantum information processing, when appropriate materials do not exist in nature or are unstable. So far there is no stable material for the Kitaev spin Hamiltonian with anisotropic interactions on a honeycomb lattice, which plays a crucial role in the realization of both Abelian and non-Abelian anyons. Here, we show two methods to dynamically realize the Kitaev spin Hamiltonian from the conventional Heisenberg spin Hamiltonian using pulse-control techniques based on the Baker-Campbell-Hausdorff (BCH) formula. In the first method, the Heisenberg interaction is changed into Ising interactions in the first process of the pulse sequence. In the next process of the first method, we transform them to a desirable anisotropic Kitaev spin Hamiltonian. In the second more efficient method, we show that if we carefully design two-dimensional pulses that vary depending on the qubit location, we can obtain the desired Hamiltonian in only one step of applying the BCH formula. As an example, we apply our methods to spin qubits based on quantum dots, in which the effects of both the spin-orbit interaction and the hyperfine interaction are estimated.

Topological quantum computation has attracted considerable interest due to its robustness to local perturbations^1^. Anyons, which obey different statistics from bosons and fermions, are also of fundamental interest in physics^2^. Kitaev^3^ provided an exactly-solvable model of a spin-1/2 system on a honeycomb lattice with potential links to topological quantum computation, for both Abelian and non-Abelian anyons. The Kitaev Hamiltonian is given by an anisotropic spin model on a 2D honeycomb lattice


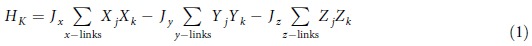


where 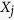, 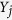 and 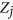 are the Pauli spin operators and the interaction type (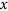, 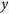, and 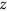 links) depends on the direction of the bond between the two sites (Fig. 1). The model in Eq. (1) can be mapped to free Majorana fermions coupled to a 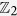 gauge field and has two types of interesting ground states, the so-called phase 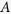 and phase 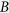, depending on the relative magnitude of 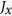, 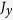 and 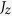. The region 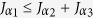, where 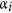 (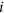 = 1,2,3) refers to 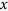,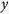,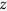, is the gapless 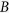 phase in which non-Abelian anyons appear, and the other region is the gapped phase 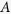, where Abelian anyon statistics is expected. In the 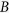 phase, an additional external magnetic field opens an energy gap. This Kitaev model has opened a new possibility of realizing anyon based on spin systems. However, it is not easy to find materials that have such anisotropic spin-spin interactions.

Even if we can find a possible material for realizing a desired Hamiltonian, we have to integrate and fabricate it by attaching many electrodes and probes to confirm whether it is sufficiently controllable^4^,^5^. Regarding artificial realizations of the Kitaev Hamiltonian, theoretical proposals have been made using optical lattices^6^,^7^ and superconducting qubits^8^. In Ref. [You]=>[8], You *et al.* used different qubit-qubit interactions depending on the coupling direction. Here, we consider how to generate the Kitaev Hamiltonian starting from the Heisenberg Hamiltonian.

The Heisenberg Hamiltonian describes two-body interactions in many magnetic materials and artificial systems such as cold atoms^6^, semiconductor quantum dot (QD) systems^9^,^10^,^11^,^12^,^13^,^14^,^15^, donor systems^16^,^17^,^18^,^19^, and nitrogen-vacancy (NV) centers^20^,^21^,^22^. The Heisenberg Hamiltonian is given by


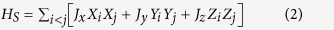


where only the nearest-neighbor interactions are assumed here. The problem to be solved is to find a way to derive the Kitaev Hamiltonian Eq. (1) from the Heisenberg Hamiltonian of Eq. (2). The difficulty is to derive the anisotropic interaction of the Kitaev Hamiltonian from the uniform interaction of the Heisenberg Hamiltonian. For example, although the tunneling couplings 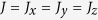 of the spin qubits based on QDs can be varied uniformly by attaching gate electrodes, we cannot control the anisotropy of the interactions by only changing the strength of the tunneling couplings^23^. The purpose of this paper is to propose two methods to dynamically derive the Kitaev Hamiltonian from the Heisenberg Hamiltonian.

The two methods provided here are based on pulse-control technique using the Baker-Campbell-Hausdorff (BCH) formula between the Heisenberg Hamiltonian and the transformed Hamiltonians. These transformed Hamiltonians are produced by applying appropriate pulse sequences to the Heisenberg Hamiltonian. The BCH formula is useful for creating desirable effective Hamiltonians^24^. However, because unwanted terms are generated by the BCH formula, it is desirable to reduce the number of times the BCH formula is applied to different transformed Hamiltonian.

In the first method provided here, which we call the direct method, the Heisenberg interaction is changed into 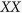, 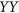 and 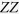 Ising couplings, by using the corresponding transformed Hamiltonians in the first process. In the second process of the direct method, three other transformed Hamiltonians are used to change the Ising couplings into the desired 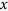, 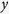 and 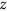-links. When we count the number of the steps required to obtain the different transformed Hamiltonians, the direct method requires six steps to obtain the Kitaev spin Hamiltonian. In the second method, which we call the efficient method, we show that if we carefully design two-dimensional (2D) pulses that vary depending on the qubit location, we can obtain the desired Hamiltonian in only one step where one transformed Hamiltonian is used.

Because the engineered Hamiltonian is effective only for a finite time interval, the dynamical approach requires a *refresh* process in which the same pulse sequence for generating the Kitaev Hamiltonian is carried out. The idea of repeating the production process is very common in conventional digital computers, such as dynamic random access memory (DRAM), which essentially is a big capacitor and the amount of electric charge is lost over time^25^. We consider the refresh overhead of the dynamical methods, and compare the two proposed methods quantitatively.

As a concrete example of the application of our methods, we consider the spin qubits based on QDs. In general, QDs have both spin-orbit interactions^26^,^27^,^28^ and hyperfine interactions^29^,^30^,^31^. Therefore, we will discuss the effects of these interactions, other than the unwanted terms derived from the BCH formula, on the topological Hamiltonian, focusing on the gapped phase (phase 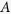).

## Results

### Dynamical creation of the Kitaev spin Hamiltonian from the Heisenberg model

Now, we explain how to derive Eq. (1) from the Heisenberg Hamiltonian Eq. (2). The “creation” of Eq. (1) is carried out by combining 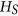 with a transformed Hamiltonian 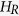, which is produced by applying a customized pulse sequence to 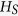, like in nuclear magnetic resonance (NMR), by a repeated application of the BCH formula. Concretely, the target Hamiltonian 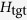 is approximately obtained by 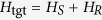, such as


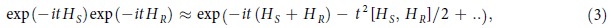


when 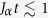. In the exponent of the right side of this equations, the terms 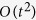 beyond the first term are the unwanted ones. The transformed Hamiltonian 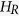 is produced by using rotations of the Pauli operators, 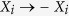, 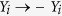 and 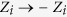. These rotations are obtained by the operations given by


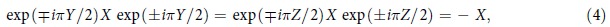


and 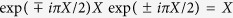. We have similar equations for the rotations of the 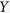 and 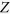 operators. In the following, we show that the first method (direct method) uses Eq. (3) six times, but the second method (efficient method) uses it only once.

## Direct method

**Figure 2:**
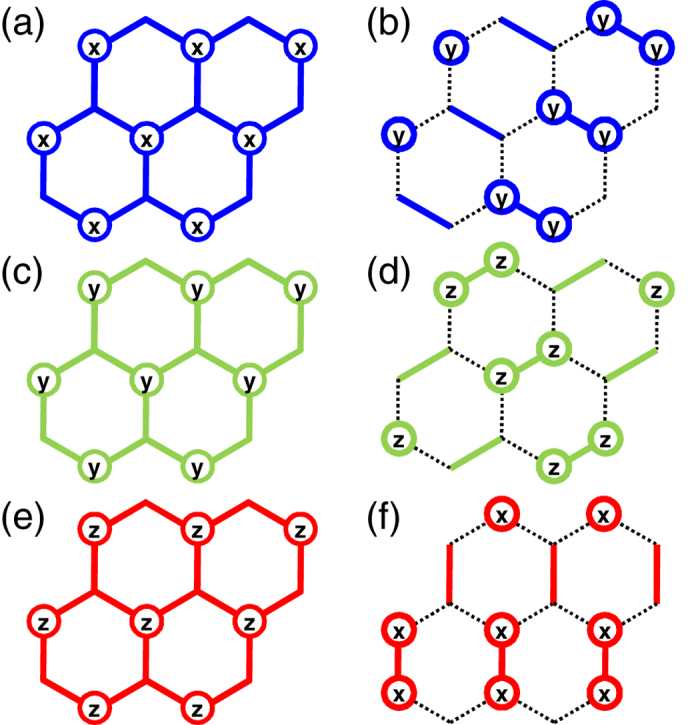
Direct method to dynamically produce a Kitaev Hamiltonian from the Heisenberg model. The symbols 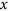, 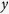 and 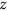 in the lattice sites show the application of 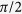-pulses around 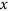, 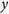 and 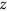, respectively. The bonds with dotted lines indicate that there is no interaction between the connected sites. (**a**) Pulse mapping of 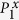 to create the Ising Hamiltonian, 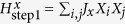 in 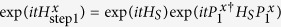. (**b**) Pulse mapping to select only the 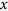-link of the Kitaev Hamiltonian from the Ising Hamiltonian of (**a**). (**c**) and (**e**) express pulse distributions for generating 
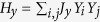
 and 
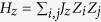
, respectively. (**d**) and (**f**) show pulse pattern to select only the 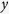 and 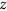 links, respectively.

The direct way to convert Eq. (2) to Eq. (1) requires six steps, as shown in Fig. 2. The first process is to create the three Ising Hamiltonians, 
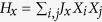
, 
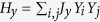
, 
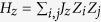
, from Eq. ((2)) ==> (2) as shown in Figs. 2(a,c,e). The generated Ising Hamiltonians are described by


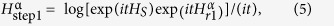


where 
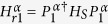
 (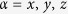) is a rotated Hamiltonian, in which 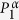 shows 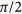-pulse rotations around the 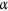-axes on the lattice sites of Figs. 2(a,c,e). The next process is to eliminate unnecessary Ising interactions, such as


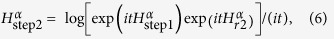


where 
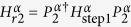
 is obtained by applying a 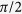-pulse operation 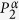 depending on the links in Figs. 2(b,d,f).

Thus, the Kitaev Hamiltonian is dynamically obtained by


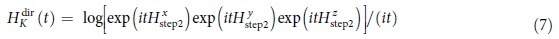


(See also Sec. I of the supplementary information). Note that parts of the Kitaev Hamiltonian do not commute, 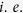, 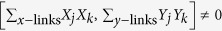. Therefore, the unwanted terms emerge even in the process Eq.((7)) ==> (7) of combining the 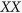, 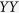 and 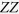 Ising couplings. In the following, we show a better method in which the 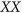, 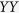 and 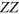 Ising couplings are generated in a single process.

## Efficient method.

**Figure 3:**
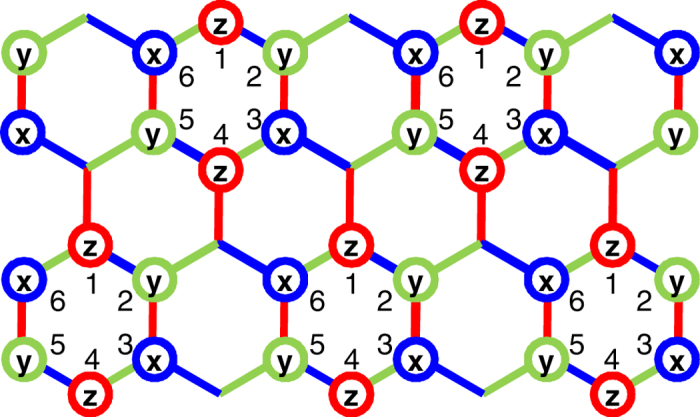
Efficient method to dynamically produce a Kitaev Hamiltonian from the Heisenberg model. The *efficient* pulse distribution 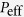 for 
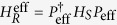
, in order to dynamically produce a Kitaev Hamiltonian from the Heisenberg model via one step. The 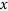, 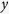 and 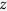 on the lattice sites show the application of 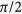-pulses around 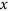, 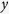 and 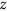, respectively.

Because the coherence time is limited, a generation method using less time can be regarded as more efficient. When we apply rotation pulses more compactly, the Kitaev Hamiltonian 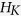 is produced more efficiently from 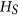. Fig. 3 shows the distributions of the rotation pulses 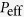 by which the BCH formula is used only once, such that


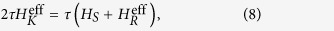


with 
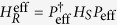
. The 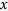-link of the colored honeycomb is produced by applying a rotation around the 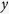-axis and that around the 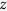-axis on both sides of the link. Similarly, the 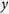 (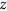)-link is produced by a rotation around the 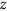 (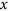)-axis and around the 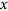 (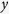)-axis on both sides of the link (see also Sec. II of the supplementary information).

## Refresh overhead

If 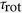 denotes the time of a single-qubit rotation, it takes 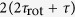 and 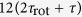 to produce the rotations 
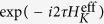
, and 
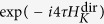
, respectively. Similar to the conventional DRAM, here we define the *refresh overhead* as the effectiveness of the refresh of the quantum state:


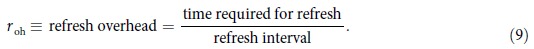


The refresh overhead of the efficient method presented above is


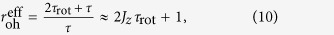


and that of the direct method shown previously is


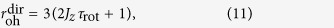


for 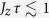. Thus, the efficient method is three times more efficient than the direct method.

## Fidelity

Let us numerically estimate the improvement of the efficient method by calculating a *time-dependent gate fidelity*^32^. The time-dependent gate fidelity is defined by


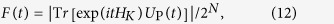


where 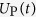 denotes the evolution operator of the pulsed system. The gate fidelity shows how well the transformed Hamiltonian evolves compared with 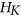. For the direct method, 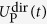 is given by


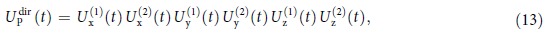


with 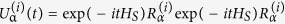, for 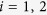 and 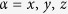. Note that 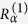 and 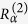 transform 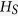 into the rotated ones shown in Figs. 2(a,c,e) and (b,d,f), respectively. In contrast, for the efficient pulse arrangement, 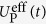 is expressed by


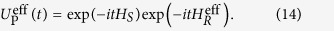


**Figure 4:**
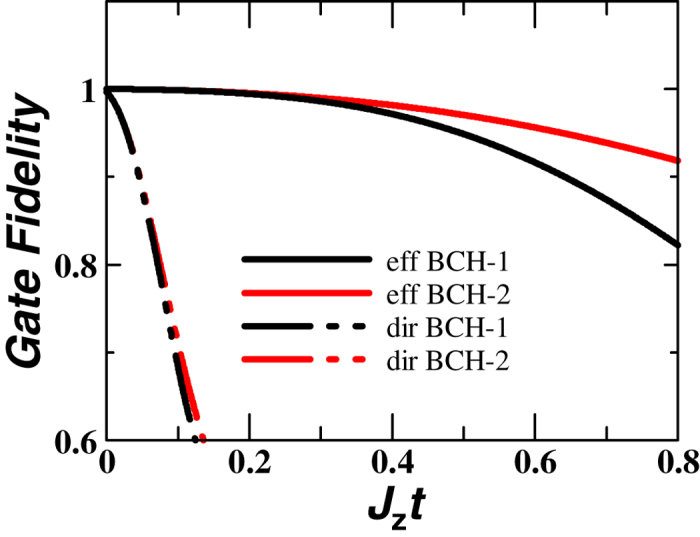
Numerically-calculated gate fidelity. Here “dir” corresponds to the 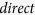 method (Fig. 2), and “eff” corresponds to the *efficient* method (Fig. 3), respectively. “BCH–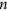
” means that the BCH formula is applied 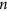 times. Repeatedly applying the BCH formula corresponds to a *refresh process*, which improves the gate fidelity. *J_x_* = *J_y_* = 0.30 *J_z_*, *d*_α_ = 0.1 and δ*h*_α_ = 0.1.

Thus, the evolution operator Eq. (14) of the efficient method is much simpler than that of the direct method Eq. (13). Here we consider the general case in which the spin-orbit interaction and the hyperfine interaction are added to the Heisenberg Hamiltonian Eq. (2), assuming the spin qubits based on QDs. The spin-orbit interaction is expressed by


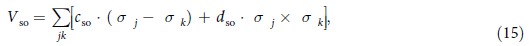


where 
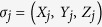
, and the magnitudes of the spin-orbit vectors 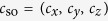 and 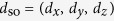 are 10^−2^ smaller^28^ than 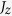. The hyperfine interaction is given by the fluctuation of the field^30^, such as 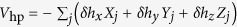. We treat the hyperfine field as a static quantity because the evolution of the hyperfine field is ~10 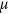
s and much slower than the time-scale of^11^ the pulse-control ~100 ns. The total Hamiltonian of this system is 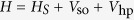. The Chebyshev expansion method is used for calculating the time-dependent behavior until its 6th-order term^33^. We have considered several parameter regions such as (i) 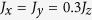, 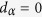, and 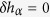, (ii) 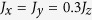, 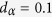, and 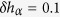, and (iii) 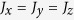, 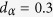, and 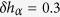 (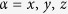). Figure 4 shows for the numerical results for 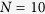 qubits (two honeycomb lattices) for *J_x_* = *J_y_* = 0.3 *J_z_*, *d*_α_ = 0.1 and δ*h*_α_ = 0.1. In various parameter regions, the overlap with the Kitaev Hamiltonian is excellent when using the pulse-controlled method. We also find that iterating the same BCH formula^34^ greatly increases the gate fidelity, which is similar to the bang-bang control^35^.

## Time-dependent Eigenvalue

**Figure 5:**
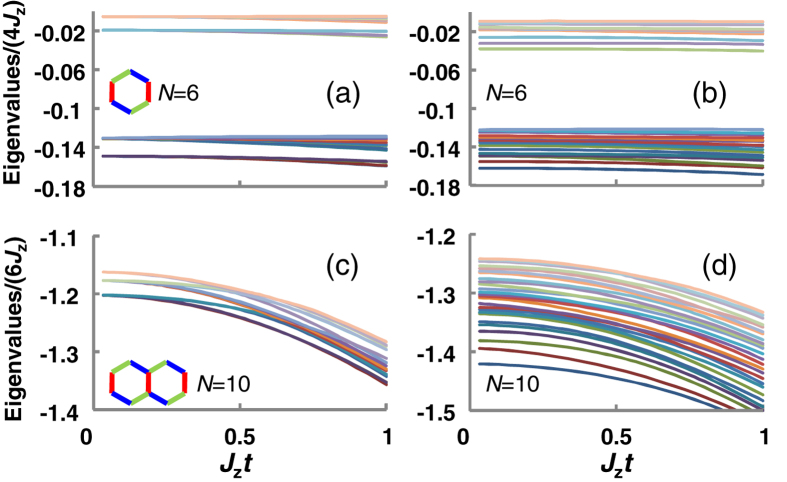
Time-dependent eigenvalues of the effective Hamiltonian. Time-dependent eigenvalues of the effective Hamiltonian 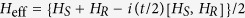, for 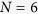 (**a,b**) and 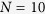 (**c,d**). 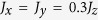 (a,c) use 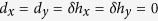. (b,d) use 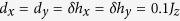. Eigenenergies are scaled by 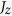.

In order to directly see the effects of the unwanted terms, spin-orbit terms, and hyperfine terms, we calculate the time-dependent eigenvalues of the effective Hamiltonian 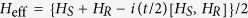, of the efficient method. Because of limited computational resources, we show the numerical results for 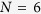 and 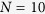. We find that an energy gap opens up in the 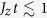 region. The energy gap becomes narrow for 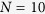, compared for 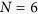, because of finite-size effects. When we compare Fig. 5(d) with Fig. 5(c), we find that the spin-orbit terms and the hyperfine terms decrease the energy gap for large-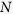 systems. In many systems, we cannot neglect additional interactions other than the Heisenberg interactions. Here, the spin-orbit and the hyperfine interactions represent such additional interactions. The results of Figs. 5(b,d) show that, although the energy gap between the ground state and the excited state of Kitaev Hamiltonian is modified by those interactions, the energy gap is detectable as long as the effect of the additional interactions is small.

## Toric code Hamiltonian

The unperturbed Hamiltonian of the 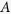 phase is given by 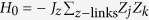, whose ground state is a degenerate dimer state. Then, 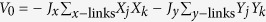 acts as a perturbation and generates the toric code Hamiltonian^3^,^36^. Comparing the unwanted terms 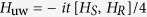, the spin-orbit terms, and the hyperfine terms, with the effective toric code Hamiltonian in Ref.^3^ implies the constraints


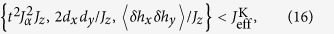


where 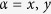 and 
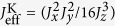
 (see Sec. VI of the supplementary information). From these estimates, in order to realize the topological quantum computation, both the spin-orbit and the hyperfine interactions should be as small as possible.

## Effects of errors in rotations.

Let us consider the effects of errors in rotations. In Eq. (4), when there is a pulse error 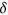 in 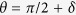. These rotations are carried out by the operations:


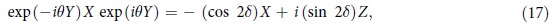



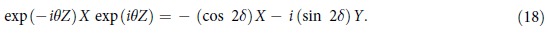


We have similar equations for the 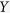 and 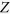 components. When we apply these equations with 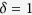 to the Heisenberg Hamiltonian 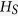, we have


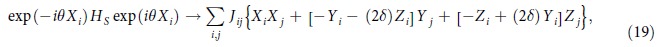



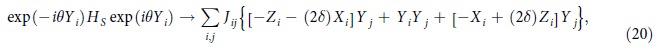



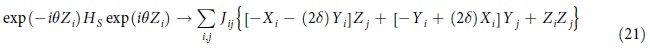


Thus, the unwanted terms from the pulse errors are counted in order of 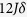. It is considered that these pulse errors can be reduced by using the conventional composite pulse method developed^37^,^38^ in NMR.

## Discussion

Because our methods use many single-qubit rotations, a short 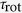 is important such that all the operations can be carried out during the coherence time of the system. For example, when 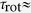 1 ns, the efficient method requires a time of 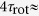 4 ns and the direct method requires a time of 24 ns. In this case, the coherence time should be longer than at least 24 ns for the realization of both methods. As an example, we consider the spin-qubit based on QDs, in which the coherence time is estimated by the dephasing time. When the dephasing time is 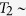 10 ns as in Ref. [11], only the efficient method is applicable. When the dephasing time is 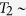 100 ns as in Ref. [12], both methods can be applied.

Next, let us discuss a measurement process of topological quantum computation in spin qubits. The toric code and the surface code are based on the stabilizer formalism^39^,^40^ where desired quantum states are obtained by stabilizer measurements. These measurements can be carried out using conventional spin-qubit operations by manipulating the Heisenberg model with appropriate magnetic fields. However, because the desired states are not always eigenstates of the Heisenberg Hamiltonian, the desired states are not preserved^41^. Thus, our proposed methods, which can preserve the desired states of the topological quantum computation, are important after the measurement. Let us estimate the measurement time in more details. In each measurement process of the surface code, four CNOT gates and two Hadamard gates are required^39^. When each CNOT gate consists^10^ of two 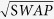
s and each 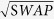 requires a time 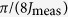, where 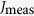 is a Heisenberg coupling strength for the measurement, one stabilizer measurement cycle approximately requires a time 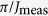. Because a short measurement time and a long coherence-preserving time 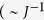) are preferable, it is desirable for the coupling strength between qubits to be changeable, and therefore 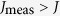 is desirable. The coupling 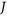 of the Heisenberg interaction can be changed by the gate voltage in spin-qubit systems based on QDs.^11^,^12^,^13^,^14^. As an example, 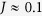-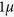
eV is obtained, when the voltage difference between two GaAs QDs is less than 10 mV^11^, and we can choose 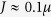
eV and 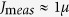
eV. When 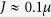
eV (= 0.0116 K), the period 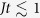 corresponds to the refresh time 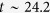 ns.

Because the fabrication process is not easy in qubits of any type, the variation of the coupling constant 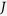 cannot be avoided in experiments. In this paper, we have shown the effect of the spin-orbit and the hyperfine interactions as examples of the unwanted terms of our methods. Thus, as long as the variation of 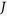 is small, it can be included as a small perturbation without significant effects on the generation of the Kitaev Hamiltonian and the toric code Hamiltonian.

In summary, we proposed two methods to dynamically generate a Kitaev spin Hamiltonian on a honeycomb lattice from the Heisenberg spin Hamiltonian by using a dynamical approach. We also considered the effects of the unwanted terms of the BCH, the spin-orbit interaction, and the hyperfine interaction, for spin qubits based on QDs. We clarified that, if these terms are sufficiently small, a dynamic topological quantum computation is available by periodically reproducing the topological Hamiltonian.

## Additional Information

**How to cite this article**: Tanamoto, T. *et al.* Dynamic creation of a topologically-ordered Hamiltonian using spin-pulse control in the Heisenberg model. *Sci. Rep.* **5**, 10076; doi: 10.1038/srep10076 (2015).

## Supplementary Material

Supplementary Information

## Figures and Tables

**Figure 1:**
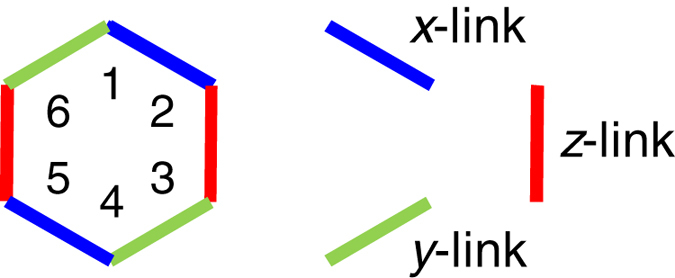
Kitaev model on a honeycomb lattice. The Kitaev model has nearest neighbor interactions on the vertices of a honeycomb lattice, where the *x*-links have *XX* interactions, the *y*-links have *YY* interactions, and the *z*-links have *ZZ* interactions.
