# Erratum: Dynamic creation of a topologically-ordered Hamiltonian using spin-pulse control in the Heisenberg model

**DOI:** 10.1038/srep18508

**Published:** 2016-01-12

**Authors:** Tetsufumi Tanamoto, Keiji Ono, Yu-xi Liu, Franco Nori

Scientific Reports 5: Article number: 1007610.1038/srep10076; published online 06172015; updated on 01122016

This Article contains typographical errors.

In Equation (1):


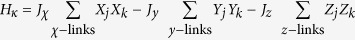


should read:


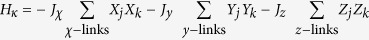


In the Introduction section,

“In Ref. [You]=>[8], You *et al.* used different qubit-qubit interactions depending on the coupling direction.”

should read:

“In Ref. [8], You *et al.* used different qubit-qubit interactions depending on the coupling direction.”

In the Direct Methods section,

“from Eq. ((2)) ==> (2) as shown in Fig. 2(a,c,e).”

should read:

“from Eq. 2 as shown in Fig. 2(a,c,e).”

And lastly,

“Therefore, the unwanted terms emerge even in the process Eq.((7)) ==> (7) of combining the *XX*, *YY*and *ZZ* Ising couplings.

should read:

“Therefore, the unwanted terms emerge even in the process Eq. (7) of combining the *XX*, *YY*and *ZZ* Ising couplings.”

