# Supplementary material for: Dynamic creation of a topologically-ordered Hamiltonian using spin-pulse control in the Heisenberg model
Source: Sci Rep. 2015 Jun 17;5:10076. doi: 10.1038/srep10076 (PMC4469982; doi:10.1038/srep10076)
Supplement: Supplementary Information [file srep10076-s1.pdf]

Supplemental material for "Dynamic creation of a topologically-ordered Hamiltonian using spin-pulse control in the Heisenberg model"

Tetsufumi Tanamoto,[1] Keiji Ono,[2] Yu-xi Liu,[3,4,5] and Franco Nori[4,6]

[1] *Corporate R & D center, Toshiba Corporation, Saiwai-ku, Kawasaki 212-8582, Japan*
[2] *Low temperature physics laboratory, RIKEN, Wako-shi, Saitama 351-0198, Japan*
[3] *Institute of Microelectronics, Tsinghua University, Beijing 100084, China*
[4] *Center for Emergent Matter Science, RIKEN, Saitama 351-0198, Japan*
[5] *Tsinghua National Laboratory for Information Science and Technology (TNList), Beijing 100084, China*
[6] *Department of Physics, The University of Michigan, Ann Arbor, Michigan 48109-1040, USA*

(Dated: February 18, 2015)

In this material, we show detailed calculations and derivations which appear in the main text.

Contents

I. Detailed explanation of the direct method 1

II. Detailed explanation of the efficient method 2

III. Unwanted terms 4

IV. Effect of spin-orbit interaction 5

V. Effect of the hyperfine interaction 6

VI. Toric code Hamiltonian and perturbation terms 7

VII. Iterative Baker-Campbell-Hausdorff (BCH) formula 8

References 8

I. DETAILED EXPLANATION OF THE DIRECT METHOD

Let us show a simple example of the derivation process of the direct method for the six qubits in Fig. 1. In Fig. 1, the x and y inside the circles show the applications of $\pi/2$-pulses around the x and y axes to the qubit of the corresponding sites, respectively. More specifically, the x inside the circle at site i indicates

$$H \rightarrow \exp[-i(\pi/2)X_i]H\exp[i(\pi/2)X_i]. \tag{1}$$

Similar operations for the y and z rotations are also applied. The Heisenberg Hamiltonian of the six qubits is given by

$$\begin{aligned} H_S &= x_{12}+y_{12}+z_{12}+x_{23}+y_{23}+z_{23}+x_{34}+y_{34}+z_{34} \\ &+ \ x_{45}+y_{45}+z_{45}+x_{56}+y_{56}+z_{56}+x_{61}+y_{61}+z_{61}, \end{aligned} \tag{2}$$

where $x_{jk} \equiv J_{jk}X_jX_k$, $y_{jk} \equiv J_{jk}Y_jY_k$, and $z_{jk} \equiv J_{jk}Z_jZ_k$. The process $H^x_{r1} = P_1^{x\dagger} H_S P_1^x$, shown in Fig. 1(a), rotates the directions of the qubits 2, 4, and 6, and we obtain

$$\begin{aligned} H^x_{r1} &= x_{12}-y_{12}-z_{12}+x_{23}-y_{23}-z_{23}+x_{34}-y_{34}-z_{34} \\ &+ \ x_{45}-y_{45}-z_{45}+x_{56}-y_{56}-z_{56}+x_{61}-y_{61}-z_{61}. \end{aligned} \tag{3}$$

Thus we can generate an XX Ising interaction given by

$$\begin{aligned} H_x &\approx H_S + H^x_{r1} \\ &= \ 2x_{12}+2x_{23}+2x_{34}+2x_{45}+2x_{56}+2x_{61}. \end{aligned} \tag{4}$$

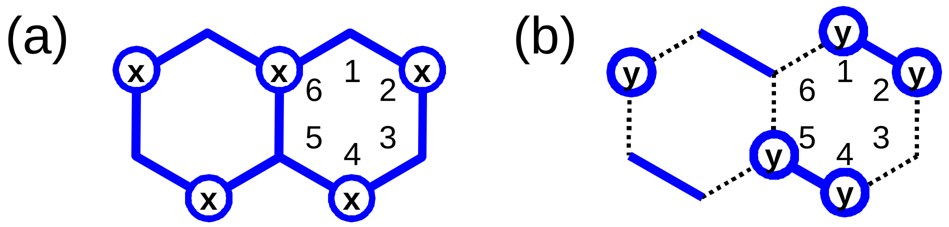

FIG. 1: Direct method to dynamically produce a Kitaev Hamiltonian from the Heisenberg model. The symbols x in the lattice sites show the application of $\pi/2$-pulses around x. The bonds with dotted lines indicate that there is no interaction between the connected sites. (a) Pulse mapping of P_1^x to create the Ising Hamiltonian, $H^x_{\rm step1} = \sum_{i,j} J_x X_i X_j$ in $\exp[itH^x_{\rm step1}] = \exp[itH_S]\exp[itP_1^{x\dagger} H_S P_1^x]$. (b) Pulse mapping to select only the x-link of the Kitaev Hamiltonian from the Ising Hamiltonian of (a). These figures are part of Fig.2 (a,b) of the main text

In the next process, P_2^x rotates the directions of the qubits 1, 2, 4, and 5 [Fig. 1(b)] around the y-axis. The transformed Hamiltonian H^x_{r2} is given by

$$H^x_{r2} = P_2^{x\dagger} H^x_{\rm step1} P_2^x \approx P_2^{x\dagger}(H_S + H^x_{r1})P_2^x, \tag{5}$$

because of $H^x_{\rm step1} = \log[\exp(itH_S)\exp(itH^x_{r1})]/(it)$. Then, we obtain

$$H^x_{r2} \;\approx\; 2x_{12} - 2x_{23} - 2x_{34} + 2x_{45} - 2x_{56} - 2x_{61}. \tag{6}$$

In this second process, the rotation can be carried out around the z-axis, instead of the y-axis. Now, we have

$$\begin{aligned} H^x_{\rm step2} &= \log[\exp(itH^x_{\rm step1})\exp(itH^x_{r2})]/(it), \\ &\approx H^x_{\rm step1} + H^x_{r2} \approx 4x_{12} + 4x_{45}. \end{aligned} \tag{7}$$

These are x-links in the ten qubits of Fig. 1 of the main text. Similarly, we can obtain y- and z-links.

II. DETAILED EXPLANATION OF THE EFFICIENT METHOD

In the efficient method to create the Kitaev Hamiltonian, the honeycomb lattice sites are divided into small units, as shown in Fig. 2 (a) and (b) in this supplemental material. In Fig. 2, the x, y and z inside the circles show the applications of $\pi/2$-pulses around the x, y and z axes to the qubit of the corresponding sites, respectively. $H_R^{\rm eff}$ is produced by applying the appropriate pulses, given by

$$H_R^{\rm eff} = P_{\rm eff}^\dagger H_S P_{\rm eff}. \tag{8}$$

Here $P_{\rm eff}$ means the product of these operations, such as

$$P_{\rm eff} = \Pi_{i,j,k}\exp[i(\pi/2)X_i]\exp[i(\pi/2)Y_j]\exp[i(\pi/2)Z_k]. \tag{9}$$

The qubit sites without the rotations are the boundary qubits of the unit of Fig. 2(b). Let us look at the twelve qubits in Fig. 2(b). The original Heisenberg Hamiltonian is expressed as

$$\begin{aligned} H_S &= x_{12} + y_{12} + z_{12} + x_{23} + y_{23} + z_{23} + x_{34} + y_{34} + z_{34} \\ &+ \; x_{45} + y_{45} + z_{45} + x_{56} + y_{56} + z_{56} + x_{61} + y_{61} + z_{61} \\ &+ \; x_{11a} + y_{11a} + z_{11a} + x_{22a} + y_{22a} + z_{22a} + x_{33a} + y_{33a} + z_{33a} \\ &+ \; x_{44a} + y_{44a} + z_{44a} + x_{55a} + y_{55a} + z_{55a} + x_{66a} + y_{66a} + z_{66a}, \end{aligned}$$

where

$$x_{jk} \equiv J_{jk}X_jX_k, \quad y_{jk} \equiv J_{jk}Y_jY_k, \quad z_{jk} \equiv J_{jk}Z_jZ_k. \tag{10}$$

When (1) $\pi/2$-pulses around the z axis are applied to the qubits 1 and 4, (2) $\pi/2$-pulses around the y axis are applied to the qubits 2 and 5, and (3) $\pi/2$-pulses around the x axis are applied to the qubits 3 and 6, the Heisenberg spin

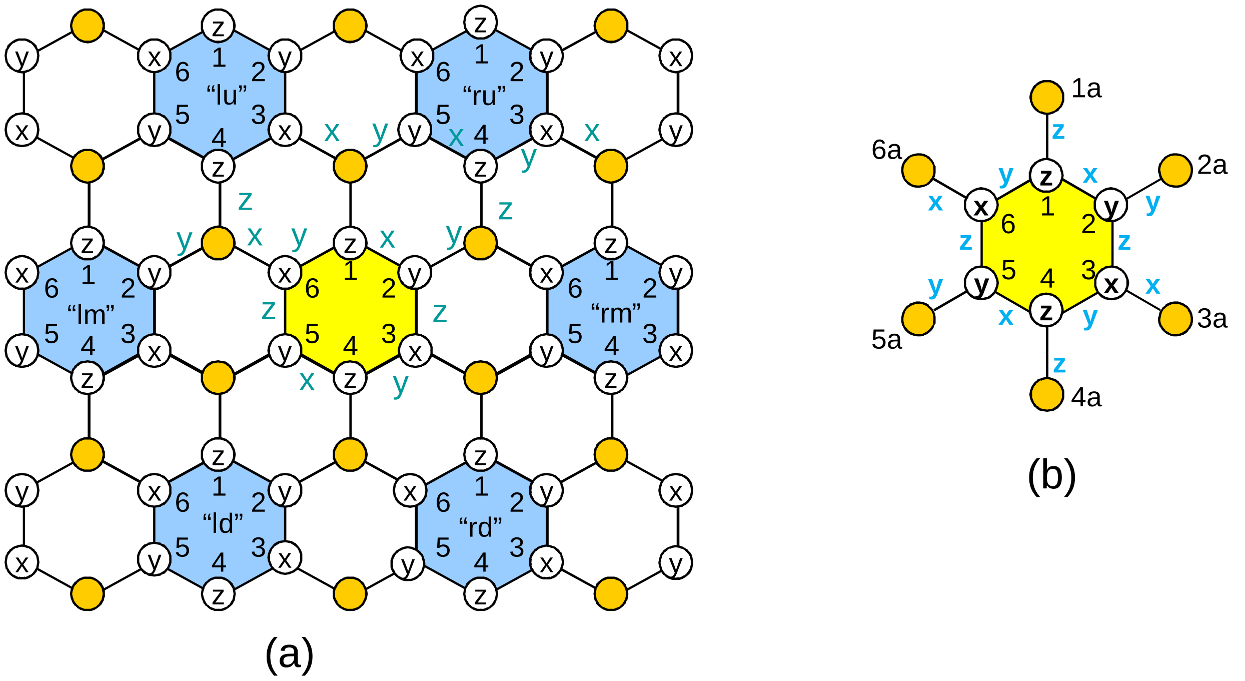

FIG. 2: The efficient pulse distribution $P_{\rm eff}$, for $H_R^{\rm eff} = P_{\rm eff}^{\dagger} H_S P_{\rm eff}$, in order to dynamically produce, via one step, a Kitaev Hamiltonian[1] from the Heisenberg model. The x, y and z on the lattice sites show the application of π-pulses around x, y and z, respectively. (a) A honeycomb lattice. (b) A unit of the transformation.

Hamiltonian H_S is changed into the rotated Hamiltonian H_R given by

$$\begin{aligned} H_R = & \; x_{12} - y_{12} - z_{12} - x_{23} - y_{23} + z_{23} - x_{34} + y_{34} - z_{34} \\ & + x_{45} - y_{45} - z_{45} - x_{56} - y_{56} + z_{56} - x_{61} + y_{61} - z_{61} \\ & - x_{11a} - y_{11a} + z_{11a} - x_{22a} + y_{22a} - z_{22a} + x_{33a} - y_{33a} - z_{33a} \\ & - x_{44a} - y_{44a} + z_{44a} - x_{55a} + y_{55a} - z_{55a} + x_{66a} - y_{66a} - z_{66a}. \end{aligned} \tag{11}$$

By applying Eq. (45), we obtain the Kitaev Hamiltonian of the unit cell, shown in Fig. 2(b), given by

$$H_K = H_S + H_R, \tag{12}$$

such as

$$\begin{aligned} H_K \;=\; & 2[x_{12} + z_{23} + y_{34} + x_{45} + z_{56} + y_{61} \\ & + z_{11a} + y_{22a} + x_{33a} + z_{44a} + y_{55a} + x_{66a}]. \end{aligned} \tag{13}$$

III. UNWANTED TERMS

The BCH formula generates the unwanted terms $H_{\rm uw}^{\rm BCH}$. Here we show the concrete form of this term of the unit of Fig. 2(b) above:

$$
\begin{aligned}
[H_S, H_R] &= [H_{12}, K^z_{23}] + [H_{23}, K^x_{12}] + [H_{12}, K^y_{61}] + [H_{61}, K^x_{12}] \\
&+ [H_{12}, K^z_{11\rm a}] + [H_{11\rm a}, K^x_{12}] + [H_{12}, K^y_{22\rm a}] + [H_{22\rm a}, K^x_{12}] \\
&+ [H_{23}, K^y_{34}] + [H_{34}, K^z_{23}] \\
&+ [H_{23}, K^y_{22\rm a}] + [H_{22\rm a}, K^z_{23}] + [H_{23}, K^x_{33\rm a}] + [H_{33\rm a}, K^z_{23}] \\
&+ [H_{34}, K^x_{45}] + [H_{45}, K^y_{34}] + [H_{34}, K^x_{33\rm a}] + [H_{33\rm a}, K^y_{34}] + [H_{34}, K^z_{44\rm a}] + [H_{44\rm a}, K^y_{34}] \\
&+ [H_{45}, K^z_{56}] + [H_{56}, K^x_{45}] \\
&+ [H_{45}, K^z_{44\rm a}] + [H_{44\rm a}, K^x_{45}] + [H_{45}, K^y_{55\rm a}] + [H_{55\rm a}, K^x_{45}] \\
&+ [H_{56}, K^y_{61}] + [H_{61}, K^z_{56}] + [H_{56}, K^y_{55\rm a}] + [H_{55\rm a}, K^z_{56}] + [H_{56}, K^x_{66a}] + [H_{66\rm a}, K^z_{56}] \\
&+ [H_{61}, K^z_{11\rm a}] + [H_{11\rm a}, K^y_{61}] + [H_{61}, K^x_{66a}] + [H_{66a}, K^y_{61}] \\
&+ [H_{1\rm a3lu}, K^z_{11\rm a}] + [H_{11\rm a}, K^x_{1\rm a3lu}] + [H_{11\rm a}, K^y_{1\rm a5ru}] + [H_{1\rm a5ru}, K^z_{11\rm a}] \\
&+ [H_{1\rm a5ru}, K^x_{1\rm a3lu}] + [H_{1\rm a3lu}, K^y_{1\rm a5ru}] \\
&+ [H_{22\rm a}, K^x_{2\rm a6rm}] + [H_{2\rm a6rm}, K^y_{22\rm a}] + [H_{2\rm a4ru}, K^y_{22\rm a}] + [H_{22\rm a}, K^z_{2\rm a4ru}] \\
&+ [H_{2\rm a6rm}, K^z_{2\rm a4ru}] + [H_{2\rm a4ru}, K^x_{2\rm a6rm}], \qquad (14)
\end{aligned}
$$

where

$$
\begin{aligned}
H_{ij} &= x_{ij} + y_{ij} + z_{ij}, &(15)\\
K^x_{ij} &= x_{ij} - y_{ij} - z_{ij}, &(16)\\
K^y_{ij} &= -x_{ij} + y_{ij} - z_{ij}, &(17)\\
K^z_{ij} &= -x_{ij} - y_{ij} + z_{ij}. &(18)
\end{aligned}
$$

The 'lu', 'lm', 'ld', 'ru', 'rm' and 'rd' show the relative positions of the honeycomb lattices around the center green honeycomb lattice in Fig. 2(a). This equation can be written in a more compact manner when using equations such as

$$
\begin{aligned}
[H_{12}, K^z_{23}] + [H_{23}, K^x_{12}] &= [x_{12} + y_{12} + z_{12}, -x_{23} - y_{23} + z_{23}] + [x_{23} + y_{23} + z_{23}, x_{12} - y_{12} - z_{12}] \\
&= -2[X_1X_2, Y_2Y_3] + 2[Y_1Y_2, Z_2Z_3] \\
&= 4i\left[Y_1X_2Z_3 - X_1Z_2Y_3\right], \qquad (19)
\end{aligned}
$$

$$
\begin{aligned}
[H_{61}, K^x_{12}] + [H_{21}, K^y_{16}] &= [x_{61} + y_{61} + z_{61}, x_{12} - y_{12} - z_{12}] + [x_{21} + y_{21} + z_{21}, -x_{16} + y_{16} - z_{16}] \\
&= 2[y_{61}, -z_{12}] + 2[x_{61} + z_{61}, x_{12}] \\
&= 4i\left[Z_6Y_1X_2 - Y_6X_1Z_2\right]. \qquad (20)
\end{aligned}
$$

The unwanted terms which includes the qubits of the single honeycomb lattice are given by

$$
\begin{aligned}
&(123; z, x) = 4iJ_zJ_x[Y_1X_2Z_3 - X_1Z_2Y_3] \\
&(234; y, z) = 4iJ_zJ_y[X_2Z_3Y_4 - Z_2Y_3X_4] \\
&(345; x, y) = 4iJ_xJ_y[Z_3Y_4X_5 - Y_3X_4Z_5] \\
&(456; z, x) = 4iJ_zJ_x[Y_4X_5Z_6 - X_4Z_5Y_6] \\
&(561; y, z) = 4iJ_zJ_y[X_5Z_6Y_1 - Z_5Y_6X_1] \\
&(612; x, y) = 4iJ_xJ_y[Z_6Y_1X_2 - Y_6X_1Z_2] \\
\\
&(211_a; z, x) = 4i[Y_2X_1Z_{1_a} - X_2Z_1Y_{1_a}] \\
&(11_a5_{ru}; y, z) = 4i[X_1Z_{1_a}Y_{5_{ru}} - Z_1Y_{1_a}X_{5_{ru}}] \\
&(4_{ru}2_a2; y, z) = 4i[X_{4_{ru}}Z_{2_a}Y_2 - Z_{4_{ru}}Y_{2_a}X_2] \\
&.... \qquad (21)
\end{aligned}
$$

For example, the unwanted terms of a single honeycomb lattice [upper part of Eqs. (21)] are given by

$$H_{a1}^{(l;z,x)} = (t_0/n)J_zJ_x[Y_lX_{l+1}Z_{l+2} - X_lZ_{l+1}Y_{l+2}], \tag{22}$$
$$H_{a1}^{(l+1;y,z)} = (t_0/n)J_zJ_y[X_{l+1}Z_{l+2}Y_{l+3} - Z_{l+1}Y_{l+2}X_{l+3}], \tag{23}$$
$$H_{a1}^{(l+2;x,y)} = (t_0/n)J_xJ_y[Z_{l+2}Y_{l+3}X_{l+4} - Y_{l+2}X_{l+3}Z_{l+4}], \tag{24}$$

for $l = 1, 4$.

IV. EFFECT OF SPIN-ORBIT INTERACTION

Here we show a detailed derivation of the effect of the spin-orbit (SO) interactions. The spin-orbit interaction is described by

$$\begin{aligned} V_{\rm so} &= \sum_{jk} V_{jk}^{\rm so}, \\ V_{jk}^{\rm so} &= [\mathbf{c}_{\rm so}\cdot(\boldsymbol{\sigma}_j - \boldsymbol{\sigma}_k) + \mathbf{d}_{\rm so}\cdot\boldsymbol{\sigma}_j\times\boldsymbol{\sigma}_k] \end{aligned} \tag{25}$$

where $\boldsymbol{\sigma}_j = (X_j, Y_j, Z_j)$, and the magnitudes of the spin-orbit vectors $\mathbf{c}_{\rm so} = (c_x, c_y, c_z)$ and $\mathbf{d}_{\rm so} = (d_x, d_y, d_z)$ are 10^{-2} smaller than ${J_z}^2$. Let us first consider the effective Hamiltonian of the spin-orbit interaction in the center honeycomb lattice of Fig. 2(b). We express the spin-orbit interaction after the single-qubit rotations by $V_{\rm so}^R = \sum_{jk} V_{jk}^{R,{\rm so}}$. Then $V_{\rm so}$ and $V_{\rm so}^R$ are given by

$$\begin{aligned}
V_{12}^{\rm so} &= c_x(X_1 - X_2) + c_y(Y_1 - Y_2) + c_z(Z_1 - Z_2) + d_x(Y_1Z_2 - Z_1Y_2) + d_y(Z_1X_2 - X_1Z_2) + d_z(X_1Y_2 - Y_1X_2) \\
V_{12}^{R,{\rm so}} &= c_x(-X_1 + X_2) + c_y(-Y_1 - Y_2) + c_z(Z_1 + Z_2) + d_x(Y_1Z_2 - Z_1Y_2) + d_y(-Z_1X_2 - X_1Z_2) + d_z(-X_1Y_2 - Y_1X_2) \\
V_{23}^{\rm so} &= c_x(X_2 - X_3) + c_y(Y_2 - Y_3) + c_z(Z_2 - Z_3) + d_x(Y_2Z_3 - Z_2Y_3) + d_y(Z_2X_3 - X_2Z_3) + d_z(X_2Y_3 - Y_2X_3) \\
V_{23}^{R,{\rm so}} &= c_x(-X_2 - X_3) + c_y(Y_2 + Y_3) + c_z(-Z_2 + Z_3) + d_x(-Y_2Z_3 - Z_2Y_3) + d_y(-Z_2X_3 - X_2Z_3) + d_z(X_2Y_3 - Y_2X_3) \\
V_{34}^{\rm so} &= c_x(X_3 - X_4) + c_y(Y_3 - Y_4) + c_z(Z_3 - Z_4) + d_x(Y_3Z_4 - Z_3Y_4) + d_y(Z_3X_4 - X_3Z_4) + d_z(X_3Y_4 - Y_3X_4) \\
V_{34}^{\rm so} &= c_x(X_3 + X_4) + c_y(-Y_3 + Y_4) + c_z(-Z_3 - Z_4) + d_x(-Y_3Z_4 - Z_3Y_4) + d_y(Z_3X_4 - X_3Z_4) + d_z(-X_3Y_4 - Y_3X_4) \\
V_{11_a}^{\rm so} &= c_x(X_1 - X_{1_a}) + c_y(Y_1 - Y_{1_a}) + c_z(Z_1 - Z_{1_a}) + d_x(Y_1Z_{1_a} - Z_1Y_{1_a}) + d_y(Z_1X_{1_a} - X_1Z_{1_a}) + d_z(X_1Y_{1_a} - Y_1X_{1_a}) \\
V_{11_a}^{R,{\rm so}} &= c_x(-X_1 - X_{1_a}) + c_y(-Y_1 - Y_{1_a}) + c_z(Z_1 - Z_{1_a}) + d_x(-Y_1Z_{1_a} - Z_1Y_{1_a}) + d_y(Z_1X_{1_a} + X_1Z_{1_a}) \\
&+ d_z(-X_1Y_{1_a} + Y_1X_{1_a}) \\
V_{22_a}^{\rm so} &= c_x(X_2 - X_{2_a}) + c_y(Y_2 - Y_{2_a}) + c_z(Z_2 - Z_{2_a}) + d_x(Y_2Z_{2_a} - Z_2Y_{2_a}) + d_y(Z_2X_{2_a} - X_2Z_{2_a}) + d_z(X_2Y_{2_a} - Y_2X_{2_a}) \\
V_{22_a}^{R,{\rm so}} &= c_x(-X_2 - X_{2_a}) + c_y(Y_2 - Y_{2_a}) + c_z(-Z_2 - Z_{2_a}) + d_x(Y_2Z_{2_a} + Z_2Y_{2_a}) + d_y(-Z_2X_{2_a} + X_2Z_{2_a}) \\
&+ d_z(-X_2Y_{2_a} - Y_2X_{2_a}) \\
V_{33_a}^{\rm so} &= c_x(X_3 - X_{3_a}) + c_y(Y_3 - Y_{3_a}) + c_z(Z_3 - Z_{3_a}) + d_x(Y_3Z_{3_a} - Z_3Y_{3_a}) + d_y(Z_3X_{3_a} - X_3Z_{3_a}) + d_z(X_3Y_{3_a} - Y_3X_{3_a}) \\
V_{33_a}^{\rm so} &= c_x(X_3 - X_{3_a}) + c_y(-Y_3 - Y_{3_a}) + c_z(-Z_3 - Z_{3_a}) + d_x(-Y_3Z_{3_a} + Z_3Y_{3_a}) + d_y(-Z_3X_{3_a} - X_3Z_{3_a}) \\
&+ d_z(X_3Y_{3_a} + Y_3X_{3_a})
\end{aligned}$$

Thus, the effective Hamiltonian of the spin-orbit interaction in the center honeycomb lattice is given by

$$\begin{aligned}
H_{\rm eff,center}^{\rm so} &= 2d_x(-Z_6Y_1 - Z_1Y_2 - Z_2Y_3 - Z_3Y_4 - Z_4Y_5 - Z_5Y_6 + Y_1Z_2 + Y_4Z_5) \\
&+ 2d_y(-X_6Z_1 - X_1Z_2 - X_2Z_3 - X_3Z_4 - X_4Z_5 - X_5Z_6 + Z_6X_1 + Z_3X_4) \\
&+ 2d_z(-Y_6X_1 - Y_1X_2 + [X_2Y_3 - Y_2X_3] - Y_3X_4 - Y_4X_5 + [X_5Y_6 - Y_5X_6])
\end{aligned} \tag{26}$$

Similarly, the effective Hamiltonian of the spin-orbit interactions in the honeycomb lattice of the right-up (ru), left-up (lu), and right-middle (rm) positions are given by

$$\begin{aligned}
H^{\rm so}_{\rm eff,ru} &= 2d_x(-Z_2Y_1+Y_2Z_1-Z_1Y_{1_a}-Z_{1_a}Y_{5_{ru}}-Z_{5_{ru}}Y_{4_{ru}}+Y_{5_{ru}}Z_{4_{ru}}-Z_{4_{ru}}Y_{2_a}-Z_{2_a}Y_2)\\
&+ 2d_y(Z_2X_1+Z_1X_{1_a}+Z_{5_{ru}}X_{4_{ru}}+Z_{4_{ru}}X_{2_a})\\
&+ 2d_z(X_2Y_1+X_{1_a}Y_{5_{ru}}+X_{5_{ru}}Y_{4_{ru}}+X_{2_a}Y_2), && (27)\\
H^{\rm so}_{\rm eff,lu} &= 2d_x(Y_1Z_6+Y_{6_a}Z_{4_{lu}}+Y_{4_{lu}}Z_{3_{lu}}+Y_{1_a}Z_1)\\
&+ 2d_y(-X_1Z_6+Z_1X_6-X_6Z_{6_a}-X_{6_a}Z_{4_{lu}}-X_{4_{lu}}Z_{3_{lu}}+Z_{4_{lu}}X_{3_{lu}}-X_{3_{lu}}Z_{1_a}-X_{1_a}Z_1)\\
&+ 2d_z(X_1Y_6+X_6Y_{6_a}+X_{4_{lu}}Y_{3_{lu}}+X_{3_{lu}}Y_{1_a}) && (28)\\
H^{\rm so}_{\rm eff,rm} &= 2d_x(Z_{5_{rm}}Y_{6_{rm}}+Z_2Y_3+Y_2Z_{2_a}+Y_{5_{rm}}Z_{3_a})\\
&+ 2d_y(X_2Z_3+Z_{6_{rm}}X_{5_{rm}}+Z_{2_a}X_{6_{rm}}+Z_{3_a}X_3)\\
&+ 2d_z(-Y_3X_2+X_3Y_2-Y_2X_{2_a}-Y_{2_a}X_{6_{rm}}-Y_{5_{rm}}X_{3_a}-Y_{3_a}X_3-Y_{6_{rm}}X_{5_{rm}}+X_{6_{rm}}Y_{5_{rm}}). && (29)
\end{aligned}$$

These are independent of the center honeycomb lattice, which can be understood by considering the transformation given by $1\to5$, $2\to4$, $2_a\to3$, $4_{ru}\to2$, $5_{ru}\to1$, and $1_a\to6$. By this transformation, for example, $H^{\rm so}_{\rm eff,ru}$ is written by

$$\begin{aligned}
H^{\rm so}_{\rm eff,ru} &\Rightarrow 2d_x(-Z_4Y_5+Y_4Z_5-Z_5Y_6-Z_6Y_1-Z_1Y_2+Y_1Z_2-Z_2Y_3-Z_3Y_4)\\
&+ 2d_y(Z_4X_5+Z_5X_6+Z_1X_2+Z_2X_3)\\
&+ 2d_z(X_4Y_5+X_6Y_1+X_1Y_2+X_3Y_4) && (30)
\end{aligned}$$

This expression is different from $H^{\rm so}_{\rm eff,center}$. Similarly, $H^{\rm so}_{\rm eff,lu}$, $H^{\rm so}_{\rm eff,rm}$ and other terms are different from $H^{\rm so}_{\rm eff,center}$. As an example, the 2nd-order perturbation result of $H^{\rm so}_{\rm eff,center}$ of a single honeycomb lattice is given by

$$H^{\rm so}_{\rm eff} = \frac{2d_xd_y}{J_z}(X_2^e+X_4^e). \tag{31}$$

V. EFFECT OF THE HYPERFINE INTERACTION

In the non-Abelian B phase without the hyperfine field, the external magnetic fields (h_x,h_y,h_z) play the role of opening the gap, where the effective Hamiltonian is expressed by

$$H^{(3)}_{\rm eff} \sim -[(h_xh_yh_z)/J^2]\sum_{jkl}X_jY_kZ_l. \tag{32}$$

Thus, the hyperfine (HF) interaction directly affects the gap of the Hamiltonian unless the applied magnetic field is sufficiently larger than the local hyperfine effective fields[3]. When we go back to the phase A, the effect of the hyperfine interaction is expressed by

$$\begin{aligned}
H^{\rm hp}_{\rm eff} &= \frac{1}{2J_z}(\delta h_{x2}\delta h_{x3}-\delta h_{y2}\delta h_{y3})X_2^e\\
&+ \frac{1}{2J_z}(\delta h_{x2}\delta h_{y3}+\delta h_{y2}\delta h_{x3})Y_2^e\\
&+ \frac{1}{2J_z}(\delta h_{x5}\delta h_{x6}-\delta h_{y5}\delta h_{y6})X_4^e\\
&+ \frac{1}{2J_z}(\delta h_{x5}\delta h_{y6}+\delta h_{y5}\delta h_{x6})Y_4^e. && (33)
\end{aligned}$$

Assuming the uniformity of the hyperfine interaction, such as $\langle\delta h_{x2}\delta h_{x3}\rangle=\langle\delta h_{y2}\delta h_{y3}\rangle$, we have

$$H^{\rm hp}_{\rm eff} = \frac{1}{J_z}\langle\delta h_{x2}\delta h_{y3}\rangle Y_2^e+\frac{1}{J_z}\langle\delta h_{x5}\delta h_{y6}\rangle Y_4^e. \tag{34}$$

VI. TORIC CODE HAMILTONIAN AND PERTURBATION TERMS

The unperturbed Hamiltonian of the A phase is given by $H_0 = -J_z \sum_{z-\text{links}} Z_j Z_k$, whose ground state is a degenerate dimer state. The Hamiltonian in the dimer state can be expressed by "effective spin operators", X^e, Y^e and Z^e, by pairing the original spin operators, such as[4]

$$\begin{aligned} &P[X\times Y]\to Y^e, \quad P[X\times X]\to X^e,\\ &P[Y\times Y]\to -X^e, \quad P[Z\times I]\to Z^e,\\ &P[Z\times Z]\to I^e. \end{aligned} \tag{35}$$

These pairs are taken between sites 2-3 and 5-6 in Fig. 2. The spins of the sites 1 and 4 are paired with the spins of another honeycomb lattice. Then, $V_0 = -J_x \sum_{x-\text{links}} X_j X_k - J_y \sum_{y-\text{links}} Y_j Y_k$ acts as a perturbation and generates an effective Hamiltonian

$$H^K_{\text{eff}} = -J^K_{\text{eff}} \sum_p Y^e_{p,4} Y^e_{p,2} Z^e_{p,1} Z^e_{p,4}, \tag{36}$$

with

$$J^K_{\text{eff}} = (J_x^2 J_y^2 / 16 J_z^3), \tag{37}$$

in their forth-order effects. Therefore, here we have to compare H^K_{eff} with the perturbation terms in the BCH formula, the spin-orbit interaction, and the hyperfine interaction in the same framework as the Kitaev perturbation theory. It is obvious that smaller magnitudes of the spin-orbit terms and the hyperfine interactions are desirable to achieve the condition that

$$J_z > \{J_x, J_y, |\vec{h}|\} > \{|\delta \vec{h}_{\text{hf}}|, c_{\text{so}}, d_{\text{so}}\}. \tag{38}$$

Here we show that we have more constraints to realize the TQC. We also consider the commutation relation with a *plaquette operator* given by

$$W_p = Z_1 Y_2 X_3 Z_4 Y_5 X_6, \tag{39}$$

where W_p commutes with the Hamiltonian Eq. (1) of the main text, and is described by $W^e_p = Z^e_1 Y^e_2 Z^e_3 Y^e_4$.

Let us start with the first-order unwanted terms in the BCH formula, given by $H_{\text{uw}} = -it[H_S, H_R]/4$. Many terms appear from these commutation relations (see Sec. III). In order to see their typical effect, here we choose the unwanted terms that originate from the single honeycomb whose six qubits are rotated (the center honeycomb in Fig. 2). These terms are explicitly expressed by Eq.(22-24). Because these terms do not commute with W_p nor the Kitaev Hamiltonian [Eq. (1) of the main text], they are unwanted terms in the topological quantum computation. The effective Hamiltonian of Eqs.(22-24) appears in the second-order perturbation given by $\langle a|H^{(2)}_{\text{eff}}|b\rangle = \sum'_j [\langle a|V|j\rangle\langle j|V|b\rangle/(E_0 - E_j)]$, which is expressed as

$$\begin{aligned} H^{\text{uw}}_{\text{eff}} \approx t^2 \{ & J_z \left[J_x^2 (X^e_1 X^e_2 + X^e_3 X^e_4) + J_y^2 (X^e_2 X^e_3 + X^e_4 X^e_1) \right] \\ + & J_x^2 J_y (2 Z^e_2 Z^e_4 - Z^e_1 X^e_2 Z^e_4 - Z^e_3 Z^e_2 X^e_4) \\ + & J_x J_y^2 (2 Z^e_2 Z^e_4 + Z^e_1 Z^e_2 X^e_4 + Z^e_3 X^e_2 Z^e_4) \}. \end{aligned} \tag{40}$$

Thus, this term commutes neither with H^K_{eff} nor with W^e_p. From this approximation, to realize a TQC, we have a constraint on the time, given by

$$t^2 J_\alpha^2 J_z < J^K_{\text{eff}}. \tag{41}$$

When $J_x = J_y$, this corresponds to $t < J_x/(4 J_z^2)$.

The effective spin-orbit terms are derived in a similar manner. As an example, the effective spin-orbit terms of the center honeycomb lattice of Fig. 2 are given by Eq. (31). The perturbation terms do not commute with H^K_{eff} nor W^e_p.

The effect of the hyperfine interaction is expressed by Eq. (34). Assuming the uniformity of the hyperfine interaction, such as $\langle \delta h_{x2} \delta h_{x3}\rangle = \langle \delta h_{y2} \delta h_{y3}\rangle$. The term $H^{\text{hp}}_{\text{eff}}$ also does not commute with H^K_{eff} nor W^e_p. From these estimates, in order to realize the TQC, both the spin-orbit and the hyperfine interactions should be small and we have the constraint

$$\{2 d_x d_y / J_z, \ \ \langle \delta h_{x5} \delta h_{y6}\rangle / J_z\} < J^K_{\text{eff}}. \tag{42}$$

VII. ITERATIVE BAKER-CAMPBELL-HAUSDORFF (BCH) FORMULA

Here we show that iterating the Baker-Campbell-Hausdorff (BCH) formula[5] decreases the effect of the unwanted terms in the BCH formula. When the desirable target Hamiltonian $H_{\rm tgt}$ is written using the original Hamiltonian $H_{\rm ori}$ and the transformed Hamiltonian H_R, such that

$$H_{\rm tgt} = H_{\rm ori} + H_R, \tag{43}$$

we can use the BCH formula effectively. For

$$A = i\tau H_{\rm ori}, \quad B = i\tau H_R, \tag{44}$$

the BCH formula iterating n times yields

$$\begin{aligned}(\exp A \exp B)^n &\approx \exp(it_0\{H_{\rm tgt} - (it_0/[2n])[H_{\rm ori}, H_R]\}) \\ &= \exp(it_0\{H_{\rm tgt} + H_{uw}^{\rm BCH}\}),\end{aligned} \tag{45}$$

where $t_0 \equiv n\tau$ is the duration of the n-pulse sequence[6]. The perturbed term $H_{\rm uw}^{\rm BCH}$ is given by

$$H_{uw}^{\rm BCH} = -i\frac{t_0}{[2n]}[H_{\rm ori}, H_R]. \tag{46}$$

Thus, as long as

$$(t_0/2n)||H_{\rm ori} - H_R|| \ll 1, \tag{47}$$

where $||A|| = [{\rm Tr}(A^\dagger A)/d]^{1/2}$ is the standard operator norm in a Hilbert space of dimension d, we can reduce the effect of $H_{\rm uw}^{\rm BCH}$. As the number n of repetitions for constant t_0 increases, this approximation becomes progressively better. For the efficient method described in the main text, $H_{\rm tgt}$ is the Heisenberg spin Hamiltonian and H_R is a rotated Hamiltonian depicted in Fig. 2.

[1] A. Kitaev, Annals of Physics **321**, 2 (2006).

[2] F. Baruffa, P. Stano, and J. Fabian, Phys. Rev. B **82**, 045311 (2010); Phys. Rev. Lett. **104**, 126401 (2010).

[3] L. Cywinski, Acta Phys. Pol. A **119**, 576 (2011); L. Cywinski, W.M. Witze and S. Das Sarma, Phys. Rev. Lett. **102**, 057601 (2009).

[4] G. Kells, A.T. Bolukbasi, V. Lahtinen, J.K. Slingerland, J.K. Pachos, and J. Vala, Phys. Rev. Lett. **101**, 240404 (2008).

[5] R.R. Ernst, G. Bodenhausen, and A. Wokaun, *Principles of Nuclear Magnetic Resonance in One and Two Dimensions* (Oxford University Press, Oxford, 1987).

[6] T. Tanamoto, Phys. Rev. A **88**, 062334 (2013).
